# Sex differences in the Simon task help to interpret sex differences in selective attention

**DOI:** 10.1007/s00426-016-0763-4

**Published:** 2016-03-08

**Authors:** Gijsbert Stoet

**Affiliations:** University of Glasgow, St. Andrew’s Building, 11 Eldon Street, Glasgow, UK

## Abstract

In the last decade, a number of studies have reported sex differences in selective attention, but a unified explanation for these effects is still missing. This study aims to better understand these differences and put them in an evolutionary psychological context. 418 adult participants performed a computer-based Simon task, in which they responded to the direction of a left or right pointing arrow appearing left or right from a fixation point. Women were more strongly influenced by task-irrelevant spatial information than men (i.e., the Simon effect was larger in women, Cohen’s *d* = 0.39). Further, the analysis of sex differences in behavioral adjustment to errors revealed that women slow down more than men following mistakes (*d* = 0.53). Based on the combined results of previous studies and the current data, it is proposed that sex differences in selective attention are caused by underlying sex differences in core abilities, such as spatial or verbal cognition.

## Introduction

Evolutionary psychological theories have been successful in explaining sex differences in a variety of cognitive abilities (for a comprehensive review, see Geary, 2010), including the well documented sex differences in spatial abilities and in verbal abilities (for a review of both these sex differences, see Halpern, 2012). An important feature of evolutionary psychology is the assumption that psychological mechanisms are the result of a cross-generational natural selection process (Buss, 1995). For example, men’s stronger spatial skills can be explained as resulting from the fact that ancestral promiscuous men who ranged further (which required spatial skills) had more opportunities for mating (Gaulin & Fitzgerald, 1986). When new sex differences in cognitive tasks are discovered, the academic community is faced with the challenge of either proposing novel explanations or trying to apply existing models. In this context, the current study focuses on the relatively recently discovered sex differences in selective attention.

Selective attention is defined as the cognitive mechanism underlying prioritized processing of specific types of information (for reviews see Driver, 2001; Lee & Choo, 2013; Plude, Enns, & Brodeur, 1994; Trent & Davies, 2012). It is a basic and necessary component of human cognition, because it allows for the selection and processing of task-relevant information while filtering out distracting information that might trigger wrong decisions (e.g., a hunter being distracted and hitting a fellow hunter instead of the selected animal in a flock). Attentional mechanisms are separate from more basic perceptual mechanisms and they are supported by a separate set of brain regions, including frontal and parietal association areas (Kastner & Ungerleider, 2000). Currently, there are no unifying explanations of the observed sex differences in selective attention (reviewed below).

It should be pointed out that a unified explanation of the observed sex differences might not only be of theoretical relevance, but also of practical relevance. For example, disorders of attention, such as ADHD, are far more common in boys than in girls (e.g., Gaub & Carlson, 1997, although the exact extent of gender differences depends on the ADHD subtype, Biederman et al., 2002). A more coherent model of explaining sex differences in selective attention might benefit the development of intervention models, although such implications are not further discussed in this paper.

### Review of research reporting sex differences in selective attention

The first studies showing sex differences in selective attention used the Posner cueing paradigm (Posner & Cohen, 1984; Klein, 2000). This paradigm has been used to study spatial orientation and spotlight models of attention, that is, models that explain which part of visual space is being attended. There are numerous cognitive psychological studies using variations of this paradigm. Such studies rarely investigate individual or group differences. Here, the focus is on the studies that investigated and reported sex differences. In general, in these paradigms participants view a computer monitor and are instructed to press a keyboard button as soon as they detect a target on screen (e.g., a rectangle in one of two empty placeholder frames left and right of a fixation point). A task-irrelevant cue is presented shortly *before* the target stimulus (e.g., a centrally positioned arrow pointing at or pointing away from the location of the upcoming target stimulus). The main finding of studies using this type of Posner cueing paradigm is that people cannot completely ignore the cue, even though they are instructed to do so. People typically respond more quickly when the location indicated by the cue matches that of the target; this is known as the “cue-validity effect”. The explanation is that the cue draws attention to a location, and when the target appears at that location soon after, its processing will benefit from the fact that the location is already being attended. In contrast, when the time between the cue and target becomes longer than around half a second, the cue-validity effect reverses, which is known as the “Inhibition Of Return effect” (IOR, Posner & Cohen, 1984; Klein, 2000); the explanation for this latter effect is that once the brain has identified a cue as task irrelevant, an inhibitory mechanism prohibits reorienting to that same task-irrelevant location soon after. Altogether, both the cue-validity and IOR effect reflect efficient information processing strategies when dealing with spatial information. Of relevance for the current study is that sex differences have been reported in both the cue-validity and IOR effects (see below).

Bayliss et al. (2005) were the first to report that women’s cue-validity effect is larger than that of men. This finding has been replicated by at least two independent groups (Merrit et al., 2007; Alwall et al., 2010). Bayliss et al. (2005) focused on the social nature of the cues they used (not only arrows, but also faces gazing to the left or right), and argued that women might be more biased than men to automatically process social cues. Although these authors did not go into the exact reasons why women are more sensitive to social cues, the literature they cite does (e.g., Baron-Cohen, 2000). The challenge for Bayliss’ explanation is, though, that the same phenomenon has been found with geometric shapes and even words instead of social cues.

Colzato, Pratt, & Hommel, (2012) studied sex differences in inhibition of return (IOR) while also measuring estrogen levels. They found that women in the late follicular phase of the menstrual cycle (when estrogen levels were higher) showed a *larger* IOR effect than men, and larger than women not in the late follicular phase. Colzato, Pratt, & Hommel, (2012) generally concluded that there are not enough data to explain the possible function of their observed sex differences in the Posner cueing task, yet argued that sex differences in selective visual attention are not structural, but state (i.e., hormonally) dependent.

Sex differences in visual selective attention have also been found in “flanker” paradigms. While Posner cueing paradigms have often been used to address the question which and how different areas of visual space are attended, this paradigm addresses the question which information within a processing channel is being processed (Eriksen & Eriksen, 1974) and has contributed to debate about early versus late selection processes (e.g., Hübner, Steinhauser, & Lehle, 2010). In these paradigms, participants are instructed to attend and respond to centrally presented stimuli while ignoring nearby (“flanking”) stimuli (developed by Eriksen & Eriksen, 1974). In flanker paradigms, the interference between task-relevant and task-irrelevant stimuli can be measured just as in Posner cueing paradigms (although different terms are used for the conditions, such as “compatible” versus “incompatible” rather than “valid” versus “invalid”). One of the main differences between flanker and Posner cueing paradigms is the location of the stimulus that needs to be responded to. In flanker tasks, the target is centrally presented, whereas in Posner cueing tasks peripherally. Nevertheless, it has been argued that Posner and flanker paradigms involve the same set of attentional processes (Chajut & Algom, 2009).

Stoet (2011) used a flanker task in which participants were instructed to press a key if a green circle appeared at the center position (i.e., go condition) of a 3 × 3 grid and to withhold a key press when a red circle appeared at the center position (i.e., no-go condition). A flanker appeared 200 ms before the onset of the go or no-go stimulus in one of the eight grid positions around the center positions. Because the flanker always appeared *before* the go/no-go stimulus, it was very salient. Women required more training trials than men to reach a criterion level of performance, and women responded more slowly to go-stimuli preceded by an incompatible (red) flanker. The main conclusion was that women’s performance is more strongly disrupted by incompatible flankers than men’s. Similarly, Judge and Taylor (2012) found that women were more distracted by incongruent flanking words in a word-categorization task (categorizing plants versus animals). Clayson, Clawson, and Larson (2011) found a sex difference in a standard Eriksen flanker task measuring event-related potentials (ERP). In their task, participants had to respond to the middle arrow out of five arrows presented next to one another. They found that men generally performed faster in the task, but they did not find an interaction between sex and flanker interference. Despite the lack of the sex difference in a behavioral effect of flanker interference, they found important differences in the ERP profile between valid and invalid flankers: The negativity of the ERP signal was stronger in men than in women 200 ms after stimulus onset in the case of invalid flankers (N2 signal). This signal is known to be involved in processing conflicting information.

Sänger et al. (2012) used a change detection paradigm and found that women made more mistakes detecting the location of a change in stimulus luminance when they were distracted by a change in stimulus orientation at a different location.

Finally, in the Navon letter identification task (Navon, 1977, 2003) participants view large letters (global level) composed of smaller letters (local level). When they are asked to detect a letter at the local or global level, participants detect targets at the global level faster than at the local level. Gender differences have been reported in this task, although there is not much consistency. Lee et al. (2012) found that men performed generally faster in this task, and attributed this to the established gender difference in spatial processing. In contrast, Roalf et al. (2006) found no difference between responding to global and local level in men, while women responded more quickly when a target appeared at the local level. Again, in contrast, Razumnikova and Volf (2011) found no difference between detecting letters at the global or local level in women, but found that men responded more quickly to target letters at the global than at the local level. Few of these studies investigated specific interference between the local and global level. Only Kimchi et al. (2003) reported that women were more influenced by global features when having to make a decision at local level (but no overall differences were found as in some of the other studies). Thus, while it is difficult to draw strong conclusions about a gender differences in global versus local processing, the latter study found a larger interference effect in women than in men.

In all of the reviewed studies so far, at least two different objects were presented, one of which needed to be attended while the other(s) needed to be ignored. Instead of using multiple objects, selective attention can also be studied when participants need to distinguish one out of multiple features of *one object*. Here, two different types of such paradigms are shortly reviewed in regard to gender differences: The Stroop (1935) task and the Simon task (Simon & Wolf, 1963). Because these paradigms require participants to attend different visual features of the same object, they are used to address questions about selection mechanisms rather than orientation mechanisms.

In the Stroop task, participants need to name the ink color of words while ignoring the word meaning. There is one recent large study that reported that women in all age groups ranging from 24 to 81 years old showed *less interference* than men in this task (Van der Elst, Van Boxtel, Van Breukelen & Jolles, 2006). It should be noted, however, that sex differences are often not found in the Stroop task (for a review, see MacLeod, 1991, p. 184; see also “Discussion” section).

Finally, in the Simon task (Simon & Wolf, 1963), participants need to process one stimulus dimension while ignoring another one. This effect was originally viewed as a bias to respond towards the source location of an object, even if that location is uninformative to its response (Simon, 1969). Later, Hommel (1993) demonstrated that the Simon effect depends on the spatial relation between stimuli and responses and less on an attentional orientation mechanism. Thus, the Simon task has theoretically been linked to a different explanation than only to shifts of spatial attention.

In 2015, Evans and Hampson (2015) found in a relatively large study (*n* = 176) that male participants responded generally faster in the Simon paradigm, and that the interference effect between task-relevant and irrelevant features was larger in women than in men. In contrast, Christakou et al. (2009), however, did not find sex differences in a study with 63 participants. Therefore, it is difficult to draw strong conclusions. In addition, it cannot be excluded entirely that the effect as reported by Evans and Hampson (2015) was a side effect of overall speed differences.

In summary, there is now evidence that in a number of tasks in which participants need to attend one object while ignoring another separate object, women are more influenced by the irrelevant stimulus feature. Of interest is the variety of tasks under which this has been found to be the case. The effect has been found when the target has been shown peripherally or centrally, and the effect has been found with arrow cues, face cues, and words. In contrast, in the Stroop task, in which participants need to attend and name one feature of an object (its color) while ignoring another feature (its word meaning), women have been reported to be less affected by the task-irrelevant information than men.

### The current study

One of the open questions addressed in this study is whether it is the case that women are only more influenced by task-irrelevant information (i.e., distracted) when this information is present in a different object than the target object (as was the case in the Posner and flanker tasks). One of the reasons why this was considered a possibility was the study by Van der Elst et al. (2006), who found that women performed *better* than men in the Stroop task in which there is only one object.

The current study used a task based on the Simon paradigm (Simon & Wolf, 1963), in which two types of information were presented at the same time and at the same location (in that sense thus being similar to the Stroop task). If sex differences in selective attention tasks are due to a lack of focus on the task-relevant location, we should not expect a sex difference in the Simon task (given that there is only one object to attend to). If women show a larger interference in the Simon task, then a different explanation is necessary. An alternative explanation is that the effect is simply related to spatial processing; after all, all the reviewed tasks in which women were more negatively affected by task-irrelevant information required the use of spatial information to produce a response (and to disambiguate the irrelevant stimulus). If the involvement of spatial information is indeed playing a role, the same sex difference should be found in the Simon task as was previously found in flanker and Posner cueing tasks.

This study also investigated a separate effect, namely post-error adjustment; it is well established that participants, in general, adjust response speed following erroneous decision making (Rabbitt & Rodgers, 1977). Thakkar et al. (2014) recently argued that women adjust more strongly than men to errors. This effect is separate from the sex difference in selective attention and can in principle occur in any response time task. The existence of this recently discovered effect is further tested in this study.

## Methods

### Participants

Participants were recruited for participation in an online study advertised on various web sites between August 2014 and April 2015. Of the 746 participants who completed the study for the first time, participants under 18 and above the highest common age under 65 years for male and female participants (which was 53 years) were excluded. Note that the rationale for this exclusion was to make sure that any possible sex difference could not be explained due to the fact that older participants of one sex were participating—now the highest age was the same for men and women (i.e., 53 years).

**Fig. 1:**
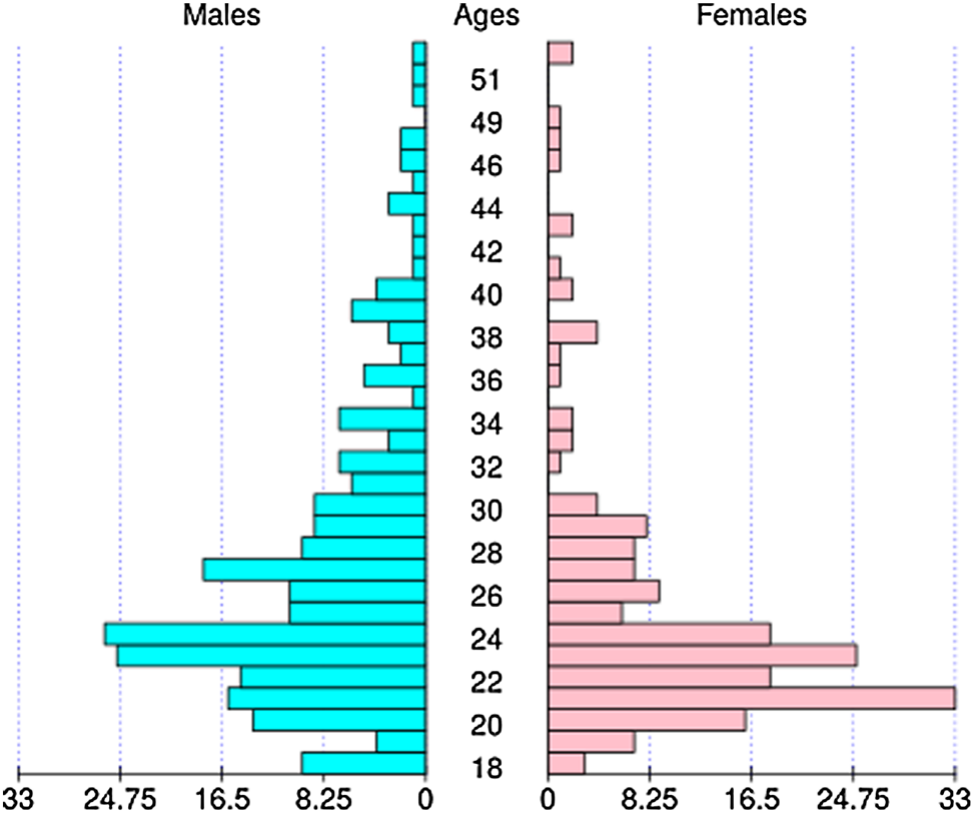
Population pyramid of the 236 male and 182 female participants. The average age of male participants (27 years) was slightly higher than that of women (25 years, *p* = .01). If participants under 23 years of age would be excluded, the difference would no longer be significantly different and conclusions drawn from the analyses would not differ, though

Participants who reported they had been disturbed during the experiment, who reported they had taken any kind of drugs that might negatively affect performance (including prescription drugs and alcohol, but not caffeine), those who reported to be very tired, and those who did not perform significantly different from performing at chance level (as tested with a proportion test on each experimental condition), and those who had not indicated their sex were also excluded. This resulted in a total number of 418 participants (236 men and 182 women, Fig. 1) from 40 different nations (as identified using the internet address, analyzed with the GeoIP database, MaxMind, Waltham, MA, USA). It should be pointed out that participant selection did not change the patterns in these findings. If the 54 participants which were excluded due to age or tiredness were included, the pattern of effects found was the same.

The study was approved by the ethics committee of the College of Social Sciences of the University of Glasgow. Informed consent was obtained from all individual participants included in the study.

### Apparatus and stimuli

This study was using online data collection based on the online version of the PsyToolkit software for programming experiments (available for free at http://www.psytoolkit.org; Stoet, 2010). The PsyToolkit website allows researchers to design, program, and setup online surveys and embed reaction time experiments in these surveys. The reaction time experiments run as Javascript applications in any modern browser without software plugins. The reaction time experiment is executed after it has been loaded into the participant’s computer, which means that the participant’s internet speed does not affect reaction times in any way. The reaction time experiment was not computationally intensive and can run reliably on standard desktop computers (for a demo, see http://www.psytoolkit.org/psychological_research_demo). The online study used the PsyToolkit option to exclude mobile devices (phones and tablets), which are known for their unreliable reaction time measurement. Online measures of reaction time measurement have generally been established as reasonably reliable by others (Crump et al., 2013; see also “Discussion” section for limitations).

Stimuli were presented in a browser window and responses measured from the regular keyboard (keys “A” and “L”). Note that on standard PC keyboard layouts, the keys “A” and “L” are on the same row of keys, with the “A” left of the midpoint of the keyboard and the “L” right of the midpoint.

The stimuli in the online response time experiment were presented in a 800 by 600 pixel area in the browser. All stimuli were colored yellow and presented on a black background. Because this was an online experiment, sizes are reported in pixels as well as the luminance if presented on a perfectly calibrated device. The target stimuli were a left pointing and a right pointing yellow arrow (144 pixels wide, 76 pixels high) which could be presented left or right from the fixation stimulus. The fixation stimulus was a yellow plus (+) symbol (48 by 48 pixels). The distance between the center of an arrow and the center of the yellow plus was 200 pixels. The fixation point and arrows were all colored in RGB value 255, 255, 0 (i.e., 100 % of both the red and green channels, which results in yellow). On a perfectly calibrated device, the luminance of stimuli was 186 cd/m^2^ and the luminance of the black background was 0.03 cd/m^2^ as measured with a Cambridge Research Systems ColorCAL on a Dell 17 in LCD monitor with standard settings under Windows XP.

### Procedure

The online study started with the presentation of text explaining the study. After consenting to participate by clicking a tickbox, participants were asked to answer a number of questions about themselves, including age and sex. People were asked how tired or fit they were (on a five point scale), and whether they were disturbed during the study (e.g., whether somebody started to talk to them), whether they could see the stimuli on screen clearly, and whether they took drugs or alcohol. Study participation lasted 11 min.

**Fig. 2:**
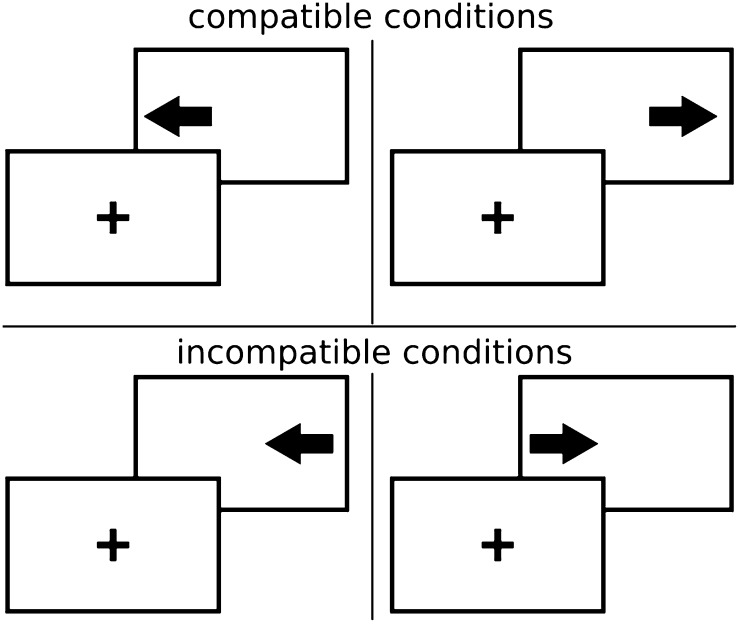
Schematic representation of the four conditions in the Simon task. Each trial started with a fixation stimulus (*plus*), followed by an *arrow* on its *left or right side*. The task was to respond with the left (A) key to a *left pointing arrow* and the right (L) key to a *right pointing arrow*. In the two instances of the *compatible* condition, the *arrow* and position relative to the fixation point matched, whereas they were in conflict in the two instances of the *incompatible* condition. It is well established that people respond more slowly in the incompatible condition, a phenomenon also known as the Simon effect

In the response time experiment part of the online session (Fig. 2), participants were instructed to respond to a left or right pointing arrow with the A or L key of their keyboard (which are left and right positioned). This resulted in stimulus–response compatible trials (i.e., when the stimulus position matched the arrow direction) and stimulus–response incompatible trials (i.e., when the stimulus position did not match the arrow direction).

Each trial lasted around 1.5 s (including stimulus presentation, response time, and short intervals between stimulus and response intervals). There were 20 training trials (not included in the data analyses) before the 102 further trials of the real data collection block. There were four conditions resulting from the position of the target stimulus (left or right of the fixation point) and the direction of the arrow.

Each trial started with the presentation of a fixation stimulus (a plus sign). The fixation stimulus was presented in six steps of 60 ms each. In the first three steps it was “growing” slightly larger, followed by three steps of shrinking. This animated fixation stimulus was intended to capture people’s attention more so than a static fixation stimulus (Abrams & Christ, 2003). Then the target stimulus (left or right pointing arrow left or right of the fixation point) was shown until a response button was pressed, but no longer than 2 s. If the wrong or no response was given within 2 s, an error message appeared for 5 s including a reminder of what the correct response should have been. A demonstration of the task and the produced data file can be tried out online: http://www.psytoolkit.org/psychological_research_demo/.

### Data analysis

All data were analyzed using the statistical software R (R development core team, 2015).

## Results

First, the variability in response times of men and women (RT) was tested. Using Bartlett’s test for comparing the variance of groups, no statistically significant differences (Bartlett’s *K*-squared = 1.65, *df* = 1, *p* = .20) between the SD of women (SD = 66 ms) and men (SD = 60 ms) were found.

**Fig. 3:**
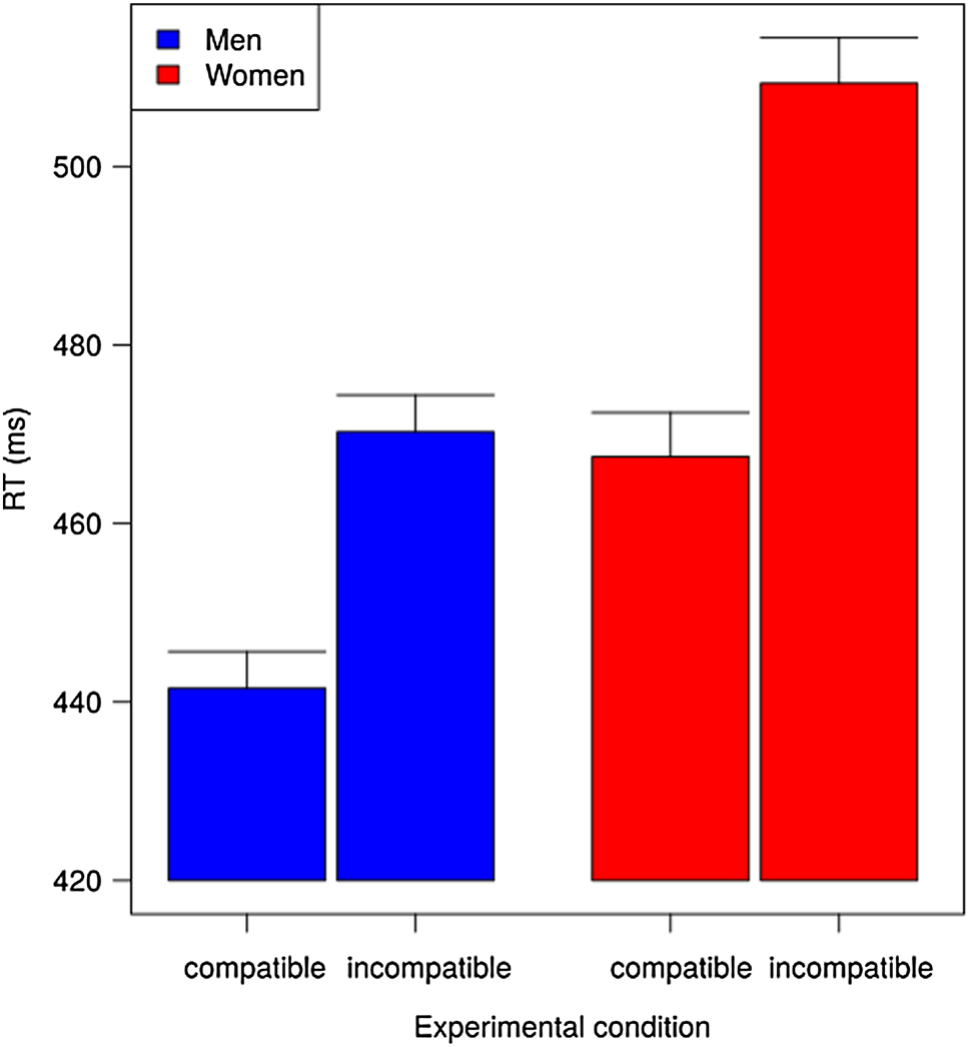
Response times as a function of sex and stimulus–response compatibility. *Bars* indicate mean + 1 SEM. The Simon effect was larger in the group of women (42 ± 2.4) than in the group of men (29 ± 2.1)

Next, the RT and error data were analyzed with an analysis of variance (ANOVA) with the within-subject factor compatibility and the between-subject factor sex (Fig. 3). The means ± 1 SEM for the different conditions will be reported. For RT analyses, error trials and those trials immediately following an error were excluded.

The well established stimulus–response compatibility (Simon) effect was confirmed, with participants (both male and female) responding 34 (SEM = 1.6) ms slower in the incompatible (487 ± 3.4) than in the compatible condition (453 ± 3.2), *F*(1,416) = 463.42, *p* < .001. Second, women performed 32 ms more slowly in the task than men, *F*(1,416) = 27.22, *p* < .001. Most important was that the Simon effect was larger in women (42 ± 2.4) than in men (29 ± 2.1), as demonstrated with the interaction between the factors sex and compatibility, *F*(1,416) = 16.51, *p* < .001. The effect size (Cohen’s *d*) of this difference was *d* = 0.39.

To check if the sex difference in the Simon effect could be related to the overall faster performance of male performance, exactly the same analysis was carried out on the normalized RT data. The rationale is as follows: Normalization (also known as standardization) adjusts the RTs such that the average RT of each participant is 0 and the SD is 1 (also known as *z* scores). By definition, this means that there are no longer any differences in overall group scores (i.e., both men and women will have an average overall RT of zero). However, because the standardization is applied to all scores, within-subject differences between conditions will still vary between subjects. Thus, we can calculate the Simon effect of the group of male and female participants after the adjustment of overall scores. Using this normalized dataset, the repeated measures ANOVA does thus not show a between-subject difference of sex, *F*(1,416) = 0.526, *p* = .47. Importantly, there was a within-subject effect of compatibility (i.e., Simon effect), *F*(1,416) = 489.483, *p* < .001, and, critically, this effect interacted with the between-subject factor sex, *F*(1,416) = 4.996, *p* = .03. This implies that the overall sex difference in speed cannot explain the sex difference in the Simon effect.

To further demonstrate that the latter conclusions about the sex difference in speed is not based on any particular statistical procedure, the following three methods were also tested. First, when the Simon effect of slow performing women was compared with that of fast responding men (where slow and fast were defined as smaller or greater than the median RT of the group), or when the Simon effect of fast performing women was compared with that of slow responding men, the Simon effect of women (41 and 42 ms, respectively) was larger than that of men (29 and 28 ms), *p*s < .01. Second, when the ANOVA was carried out on log-transformed RTs of individual participants, the effect of sex was found, *F*(1,416) = 11.92, *p* < .001. Third, when the Simon effect was regressed on overall response times (i.e., average RT of all conditions) and gender, the effect of gender was found (*p* < .0001), but no effect of overall response time (*p* = .71).

When the same ANOVA was applied to the error rates, the only statistically significant effect was that participants made more errors (3.97 ± 0.25) in the incompatible (6.03 ± 0.24) than the compatible (2.06 ± 0.13) condition, *F*(1,416) = 245.916, *p* < .001. Women’s overall error rate (3.83 ± 0.22) was not significantly different from that of men (4.21 ± 0.20), *F*(1,416) = 1.64, *p* = 0.20.

Given that female participants performed more slowly than male participants across conditions, it was further studied if it could be the case that women used a different speed-accuracy trade-off. Two different aspects of this were analyzed. First, participants’ speed was related to their accuracy (*β* = −4.2, *p* < .01), but this effect did not interact with the participant’s sex (*p* = .17). Second, for each participant the slowdown following errors was calculated. For this, for each participant who made mistakes the RTs in trials immediately following an erroneous response were compared to those following a successful response. The average slowing down in women (151 ± 13.2 ms) was more than twice as large as in men (77 ± 6.8 ms), *t*(380) = 5.3, *p* < .001. The effect size of this difference was *d* = 0.53. Note this effect does not affect the other analyses, because the first trial following an error was removed from those analyses (as is common practice in this type of study).

## Discussion

This study revealed a sex difference in the Simon task. Female participants were more strongly influenced by task-irrelevant spatial information than men. Further, responses of women were generally slower than those of men, while their accuracy levels were similar. To test if the overall slower performance of women could explain the sex difference in the Simon effect, four different methods were carried out to determine if the Simon effect remained the same. With each of these methods, the sex difference in the Simon effect was found, which means that the overall speed of participants cannot explain the finding. Further, while there were no sex differences in the speed-accuracy trade-off, post-error slowdown in women was more than double as long as in men. It is important to note that this post-error slow down did not influence the other data analyses, because the RTs of trials immediately following errors were not included in the other analyses.

Sex differences in the Simon task have not been reported before 2015, even though there have been hundreds of studies using the paradigm (as determined by the Web of Science Search engine). The lack of such effects is possible because typical cognitive psychological studies are relatively small and not designed to look for sex differences. Nonetheless, in 2015, the first study reporting a similar effect as reported here came out (Evans & Hampson, 2015). Like in the Evans and Hampson (2015) study, here it was not only found that the Simon effect was larger, but also that men responded faster than women. On the other hand, a relatively large study by Christakou et al. (2009) did not observe a sex difference in the Simon effect. It is impossible to determine why exactly the latter study did not find a sex difference in the Simon effect, but one possibility is that the study with 55 participants lacked the statistical power that the current study (*n* = 418) or that of Evans and Hampson (2015) with 176 participants had.

The findings of a sex difference in the Simon task contribute to our understanding of sex differences in selective attention. Most previous studies that found that women were more influenced by task-irrelevant stimulus information than men involved two different stimuli, whereas the current study is one of the first showing the same effect with just one stimulus. This constraints the possible range of explanations. For example, it cannot simply be the case that women have more difficulty focusing on one out of multiple objects, because here we observed the same effect even when there is just one object.

What all the studies of selective attention in which women were more influenced by task-irrelevant information have in common is that participants need to use spatial information to determine what information is relevant. In both the Posner cueing task and the flanker task, spatial information determines which stimulus to respond to while even the task-irrelevant cue sometimes has a spatial dimension. In the Simon task, the task-irrelevant location of the stimulus is a salient aspect of the stimulus. Therefore, the simplest explanation for this and previous findings is that selective attention is slowed down by subordinate processes it depends on. In the case of tasks in which spatial information needs to be disambiguated for making a decision, men will be at an advantage due to faster spatial processing. Similarly, in the case of tasks in which lexical information needs to be disambiguated, women have an advantage, for example in the Stroop task. This means that neither men nor women have an absolute advanced form of selective attention; instead, how well they perform in tasks using selective attention depends what cognitive abilities are needed to process the various stimuli involved in the task.

This simpler model is consistent with the conclusions by Colzato et al. (2012). According to these authors, sex differences in selective attention in spatial cueing tasks are dependent on hormonal fluctuations. This is what we should expect given that we know that sex hormones affect spatial cognition (e.g., in mental rotation tasks, Hausmann et al., 2000; but see a counter argument below under limitations).

The reason why sex differences are not always observed in the Stroop task needs to be addressed as well. Sex differences are not always observed in response times and error rates when the tasks are not sufficiently demanding to distinguish between men and women. As explained in the introduction, women’s advantages in verbal skills are well documented, including lexical processing (Majeres, 1999). We also know that the sex difference in verbal skills (unlike spatial skills) is considerably smaller among the best performing participants (Stoet & Geary, 2013). Therefore, we must assume that the sex difference in language skills among university students is considerably smaller than in the general population, and therefore, sex differences in linguistic tasks will be more difficult to demonstrate with university students. Indeed, most studies of the Stroop effect are carried out with university students, while the large study of Van der Elst et al. (2011), which found a sex difference, recruited participants from the population as a whole. Whether this is indeed the key factor explaining why the sex difference in Stroop interference is often not found needs to be further investigated in future studies.

### Links to biological correlates of selective attention

Given the known role of biological variables on cognitive performance (Hampson, 1990; Kimura, 1996), a fuller understanding of sex differences in selective attention will depend on studies which combine behavioral measures with biological measures. There is considerable body of research on the neurophysiological and neuroendocrinological basis of selective attention (for reviews see Pletzer, 2014; Trent & Davies, 2012). Of particular relevance for the current study is whether this research can determine whether the observed sex differences in selective attention are caused by sex differences in more basic abilities (e.g., spatial or verbal abilities), whether they may be caused by sex differences in the higher level functions (such as inhibitory mechanisms), or possibly both.

There is evidence that the female sex hormone estradiol affects inhibition in some tasks, but this evidence is still difficult to integrate in a unified model of sex differences in selective attention. For example, Colzato et al. (2010) found that while higher levels of estradiol correlated with less efficient inhibitory control in a stop-signal paradigm, higher levels of estradiol correlated with stronger levels of inhibition in an IOR paradigm (Colzato et al., 2012). They explained the differences in findings due to whether the estradiol-mediated inhibition affects input (i.e., perceptual processes in the IOR paradigm) or output processes in the stop-signal paradigm. Similar to the Colzato et al. (2010) findings, Hatta and Nagaya (2009) found that women low on estradiol (and progesterone, days 2–3 of the menstrual cylcle) were faster in reading incongruent Stroop color words than in the high-steroid mid-lutheal phase (cycle days 21–22). While Colzato et al. (2012) did not find a relation between progesterone and improved attention, Brötzner et al. (2014, 2015) reported an enhancement of attention in women with high levels of progesterone in the mid-luteal phase and hypothesize that the enhanced attention during high progesterone levels are due to the observed enhanced synchronization in the alpha frequency band in electrical cortical activity. Altogether, these studies show that there is considerable variation in levels of selective attention during the menstrual cycle. One of the challenges for the study of the role of sex hormones are the complex interactions between the different hormones, such as between progesterone and testosterone in women during the luteal phase (Pletzer et al., 2014).

In regard to the Simon task, there are different possible outcomes. If such a variation in women’s Simon effect during the menstrual cycle occurs, it can still be the case that men’s Simon effect is smaller than that of women. If that is the case, it might be that there are two independent causal pathways. On the one hand, the Simon effect might be smaller in men due to more efficient processing of spatial information, while on the other hand, this effect might become smaller when inhibitory processes in women are more efficient due to hormonal fluctuations. Alternatively, it is possible that the sex difference in the Simon effect is only due to the monthly variation (a prediction more in line with Colzato’s model of sex differences in selective attention). A study of sex differences in the Simon task while measuring sex hormone levels can answer whether this is the case.

Similarly, an important question to better distinguish between the different models would be to find out how hormones interact with the Stroop effect and negative priming effects; arguably, if sex differences in selective attention occur due to sex differences in spatial and verbal abilities, we would predict that even when women’s inhibitory control is least efficient due to hormonal fluctuations, they would still outperform men. The Hatta and Nagaya (2009) study did not include male participants, which means that their study unfortunately cannot clarify this issue.

### Limitations of the current study

In the current study, the term “attention” was used in a broad sense to incorporate the selection of relevant features and the suppression of irrelevant features in the Simon task. This type of feature-based attention is not necessarily the same as the spatial attention that is measured in the cueing task, meaning that (1) I do not mean to subscribe to the view that the Simon effect can be explained in terms of spatial attention shifts (cf. Rubicchi, Nicoletti, Iani, & Umiltà, 1997) and (2) it remains open how feature-based and location-based attention may interact (cf. Eimer, 2014).

Although the present theory is cast in terms of Simon effects (i.e., in terms of a stimulus–response [S–R] compatibility effect), the current results could have reflected a stimulus–stimulus (S–S) compatibility effect. The reason is that a left pointing arrow on the right (and a right pointing arrow on the left) was not only incompatible to the required response, but also to its position. By the same token, a left pointing arrow on the left (and a right pointing arrow on the right) was not only compatible to the required response, but also to its position. Because S–S and S–R compatibility were, thus, fully confounded and because prior research has shown that both of these effects can influence RTs (Kornblum, Hasbrouqc, & Osman, 1990), it is impossible to decide which of these factors was responsible for the current compatibility effect and its interaction with gender.

The proposed model, in essence, states that observed sex differences in selective attention result from sex differences in spatial attention and verbal abilities. Arguably, this interpretation cannot be derived from the reported data. Instead, the proposed hypothetical model is inspired by existing data, and the reported data fit that model. However, it needs further testing, for example in relation to hormonal fluctuations (see previous section).

A specific problem with the current online study is that the validity of participant’s responses cannot be verified. Further, because the experiment was presented in a browser, stimulus size and luminance will have varied between participants. It is unclear if this variation would have been similar between the two groups (men vs. women); if not, such a group difference might influence the data. And finally, participants did not answer any questions that could help to estimate levels of education or general intellect. Although it is unclear if education and intellect have a measurable effect on the Simon task, there is a possibility that if such levels affect the Simon task and that if the levels were not matched, that this could be an alternative explanation. These limitations need to be considered, although there are no reasons to assume such group differences are likely to have occurred. In this context, it should at the very least be noted that the findings of the Simon effect were very similar to the laboratory-based study of Evans and Hampson (2015).

## Conclusion and outlook

In this article, a simpler model of the observed sex differences in selective attention, including in the Simon effect, has been proposed. The model is an attempt to integrate findings from this and other studies, but the current data only support one aspect of the model, while other aspects need further testing. What is particularly needed is support for the prediction of larger interference effects in men when selective attention relies strongly on the use of verbal information. The proposed model predicts that such sex differences will occur in the Stroop task or in negative priming tasks in which there is no spatial information needed to respond (e.g., when using Stroop stimuli, Dalrymple-Alford & Budayr, 1966).
